# Effects of vitamin A supplementation in the diet of breeding geese on offspring intestinal tissue morphology and immune performance

**DOI:** 10.5713/ajas.19.0890

**Published:** 2020-03-12

**Authors:** Haiming Yang, Jingru Liang, Hang Dai, Xiaoli Wan, Zhiyue Wang

**Affiliations:** 1 College of Animal Science and Technology, Yangzhou University, Yangzhou, Jiangsu 225009, China; 2 Joint International Research Laboratory of Agriculture and Agri-Product Safety of Ministry of Education of China, Yangzhou University, Jiangsu 225009, China

**Keywords:** Vitamin A, Maternal and Offspring, Digestive Tract, Immune Performance

## Abstract

**Objective:**

The effects of maternal and offspring dietary vitamin A (VA) supplementation on early body weight, digestive tract function and immune function in goslings were studied.

**Methods:**

Yangzhou geese (180 d old) were randomly divided into 5 experimental groups of 15 females and 3 males (the males were kept until slaughter). Eggs were collected for hatching during the peak laying period. A total of 96 goslings were selected from each treatment group (each fed a basic diet supplemented with 0, 4,000, 8,000, 12,000 or 16,000 IU/kg VA) and randomly divided into 2 groups, with 6 replicates in each group and 8 goslings in each replicate. The gosling diet was supplemented with 0 or 9,000 IU/kg VA.

**Results:**

i) Villus length, villus width and the muscle thickness of the duodenum, jejunum and ileum were increased and the crypt depth was reduced after adding 12,000 IU/kg VA to the goslings’ diet (p<0.05). Adding 9,000 IU/kg VA to the offspring diet increased the length of the duodenal villi and width of the ileum and decreased the crypt depth of the ileum (p<0.05). ii) Supplementing the maternal diet with 12,000 IU/kg VA increased immune organ weight, the immune organ index and immunoglobulin content in goslings (p<0.05). The bursa weight and immunoglobulin G content of offspring were higher in the 9,000 IU/kg VA supplementation group than in the group with no supplementation (p<0.05).

**Conclusion:**

Offspring growth and development were affected by the amount of VA added into maternal diet. The negative effect of maternal VA deficiency on offspring can be compensated by adding VA to the offspring diet. Continued VA supplementation in the offspring diet after excessive VA supplementation in the maternal diet is unfavorable for gosling growth and development.

## INTRODUCTION

To date, poultry have mainly been studied in terms of their own nutritional regulation, and research on the relationship between maternal and offspring nutrition is almost nonexistent. Vitamins can act on offspring through eggs, so we studied the effect of dietary vitamin A (VA) supplementation in maternal and their offspring on the intestinal tissue morphology and immune performance of goslings. The VA can change the intestinal morphology and structure of animals [1]. The VA can improve fecundity [2] and can promote the formation of sex hormones to maintain normal bone growth and development [3–5]. The VA can also regulate the metabolism of fat, carbohydrates and proteins, promote growth, and play important roles in immune function and nonspecific responses to disease [6,7]. A lack of VA in animals will reduce mucosal immunity [8–10]; thus, immune performance is reduced, leading to various diseases [11,12]. Excessive VA can easily cause poisoning [13,14]. In our experiment, we added 0, 4,000, 9,000, 12,000 and 16,000 IU/kg VA to the diets of breeding geese. Then, we supplemented the diets of offspring with 0 or 9,000 IU/kg VA to explore the effects of VA supplementation in the maternal and offspring diets on the digestive tract and immune performance of goslings. This study will help to determine the amount of VA that can reduce the cost of geese production while simultaneously maintaining the best production performance. In addition, this study provides a reference for the ideal feed preparation method for geese in the future.

## MATERIALS AND METHODS

### Experimental design and diets

The Yangzhou University (Yangzhou, China) Animal Care and Use Committee approved all procedures in our experiments. The permit number: SYXK (Su) IACUC 2012–0029. All test geese were healthy and were obtained from the Yangzhou Geese Breeding Farm (China).

Next, a total of 90 Yangzhou geese (180-day-old) were selected and randomly distributed into 5 experimental groups with 15 female geese and 3 male geese in each group. The 5 dietary treatments included the basal diet supplemented with 0, 4,000, 8,000, 12,000, or 16,000 IU/kg VA, and the different groups of geese were fed the experimental diet for 120 d. The VA (produced by Diesman Vitamin Co., Ltd, Shanghai, China) was added in the form of 5×10^5^ IU/g acetate as active ingredient. Water and feed were provided *ad libitum*. The geese were raised in concrete pens with straw litter (2 to 3 cm thickness) and allowed to mate freely. During the whole experimental period, the breeding geese were housed under conditions of 12 h of light per day, and the room temperature was 24°C±3°C. Eggs were collected when the geese reached the age of 300 d. The eggs were hatched. A total of 96 goslings in each treatment group were randomly divided into two groups, with 6 replicates in each group and 8 goslings in each replicate. The offspring control group received only the basal diet. The VA-treated group received the basal diet supplemented with an additional 9,000 IU/kg VA (the VA supplementation dose of goslings was based on the level of VA added to the diets of domestic chicks). A basal maize-soybean meal diet was formulated to provide adequate concentrations of all the nutrients required by the geese [15] except VA, based on many years of research results from our laboratory (Table 1). The goslings were raised in concrete pens with straw litter (2 to 3 cm thickness). The offspring were fed from birth. Water and feed were provided *ad libitum*. The housing was kept clean and well ventilated. The geese were maintained on a lighting schedule of 23 h of light and 1 h of darkness per day, and the temperature was maintained at approximately 29°C. The gosling test period was 7 d. No VA was added to the basic diet premix used to feed the geese and their offspring. No VA was added to the basic diet premix of geese and their offspring.

### Sample collection and index determination

#### Intestinal tissue morphology

The goslings were sacrificed after they fed for 7 d. The duodenum, jejunum and ileum were dissected, the intestinal contents were extruded, and the contents were gently rinsed with saline (to prevent the destruction of the intestinal villus structure). The tissue samples of the duodenum, jejunum and ileum were fixed in a 4% neutral formaldehyde solution and replaced once 24 h later. Fixed 5 mm intestinal tissue sections were collected, subjected to alcohol dehydration and cleared with xylene in a JJ-12J dehydrator. After wax immersion treatment, a JB-P5 embedding machine was used for embedding. The JJ-12J dehydrator and the JB-P5 embedding machine were purchased from Wuhan Junjie Electronics Co., Ltd. (Wuhan, China). Sections (3 microns) were prepared with an RM2016 microtome that was purchased from Shanghai Leica Instrument Co., Ltd. (Shanghai, China). After staining with hematoxylin-eosin, the film was sealed with neutral gum. The villus height, villus width, recess depth and muscle thickness of the duodenum, jejunum and ileum were measured by an LY-WN-HP-SUPER-CCD image analysis system, and the ratio of the villus height to the recess depth (V/C) was calculated. A Nikon Eclipse Ci Positive Optical Microscope was used (Nikon, Shanghai, China).

The height of the villus was defined as the distance from the top of the villus to the root of the villus (lamina propria).

The width of the villus was defined as the distance at the widest part of the villus.

The depth of the crypt was defined as the distance from the root of the villus to the bottom of the intestinal gland.

Muscle thickness was defined as the vertical distance from the mucosal epithelium to the mucosal base.

#### Immune function

After sacrificing and exsanguinating the geese, the thymus, spleen and bursa of Fabricius were dissected and weighed, and the organ index was calculated. Immune organs were weighed on an electronic balance, which was produced by Beijing Sedoris Instrument System Co., Ltd. The formula used was as follows: organ index = organ weight (g)/preslaughter live weight (g)×100%. After feeding for 7 d, the 4 mL of blood was collected from the inferior vein of the goose wing. Then, the serum was separated by a DL-5M Low Speed Refrigeration Centrifuge and stored in the refrigerator until use. The centrifuge was produced by Changsha Xiangzhi Centrifuge Instrument Co., Ltd. (Changsha, China). An enzyme-linked immunosorbent assay for geese was used to determine the contents of immunoglobulin A (IgA), G (IgG), and M (IgM) in serum. The kit was purchased from Beijing Solebo Technology Co., Ltd. (Beijing, China). The reagent was prepared according to the instructions.

### Statistical analysis

The data were analyzed as part of a 5×2 factorial arrangement with five levels of breeding geese VA and two levels of gosling VA using the following statistical model. The data were subjected to analysis of variance using the general linear models procedure in SPSS 17.0 [16] and single degree of freedom orthogonal contrasts were used to partition the treatment sums of squares into their linear effects. Deviations from linearity means were determined at p<0.05.

## RESULTS

### Body weight

At 7 d of age, maternal 12,000 IU/kg VA levels had an effect on offspring body weight and weight gain (p<0.05). The weight gain of the offspring treated with 9,000 IU/kg VA was significantly higher than that of the offspring with no VA supplementation (p<0.05). Different VA levels in the maternal and offspring diets had a significant interaction effect on the weight gain of the goslings (p<0.05) [17].

### Intestinal tissue morphology

The effects of maternal and offspring dietary VA levels on the duodenal morphology of goslings are presented in Table 2. The addition of 12,000 IU/kg VA to the maternal diet significantly increased the villus length, villus width and muscle thickness and reduced the crypt depth of the offspring (p< 0.05). The length of the villi in the 9,000 IU/kg VA supplementation group was greater than that in the group with no supplementation (p<0.05). There was a quadratic relationship between the VA levels in the maternal diet and the duodenal villus width, crypt depth, and muscle thickness of the offspring (p<0.05), and there was a linear relationship with villus length (p<0.05). Different levels of VA in the maternal and offspring diets had an interaction effect on the villus length of the offspring (p>0.05).

The effect of dietary VA levels on the jejunal morphology of goslings is summarized in Table 3. The addition of 8,000 IU/kg and 12,000 IU/kg VA to the maternal diet significantly improved the villus length, villus width and muscle thickness of the offspring (p<0.05). Adding 12,000 IU/kg VA to the maternal diet significantly reduced crypt depth in the offspring (p<0.05). There was a quadratic relationship between the VA levels in the maternal diet and the jejunal villus length, villus width, crypt depth, and muscle thickness of the offspring (p<0.05). The maternal and offspring dietary VA levels had an interaction effect on the villus width, crypt depth, and muscle thickness of the offspring (p<0.05).

Table 4 shows the effect of dietary VA levels on the ileum histomorphology of maternal and their offspring. The addition of 8,000 IU/kg VA to the maternal diet significantly increased the villus length of the offspring (p<0.05). The addition of 12,000 IU/kg VA to the maternal diet significantly improved the villus width of the offspring (p<0.05). Addition of 4,000, 12,000, and 16,000 IU/kg VA to the maternal diet significantly reduced the crypt depth of the offspring (p<0.05). Adding 8,000 IU/kg VA and 12,000 IU/kg VA to the maternal significantly increased the muscle layer thickness of the offspring (p<0.05). The villus width of goslings in the 9,000 IU/kg VA supplementation group was significantly wider than that of no VA supplementation group (p<0.05), and the crypt depth of goslings in the no VA supplementation group was significantly lower than that in the 9,000 IU/kg supplementation group (p<0.05). There was a quadratic relationship between VA levels of maternal diet and villus length, villus width, crypt depth and muscle thickness of offspring (p<0.05). There was a significant interaction between the width of villus and the depth of crypt in the goslings with different VA levels of maternal and offspring (p<0.05).

### Immune function

Table 5 shows the effects of different levels of VA on thymus, spleen and bursa indices in offspring. Adding 12,000 IU/kg VA to the maternal diet significantly increased the thymus, spleen and bursa indices of the offspring (p<0.05). The thymus index of the offspring in the group with no VA supplementation was significantly higher than that in the 9,000 IU/kg supplementation group (p<0.05). There was a linear relationship between maternal VA levels and the thymus and bursa indices of offspring (p<0.05). There was a significant interaction between the maternal VA level and the offspring VA level in the thymus, spleen and bursa indices (p<0.05).

The effects of the different levels VA on the Ig contents of the offspring in serum is showed in Table 6. The IgA content in offspring with 12,000 IU/kg VA supplemented in the maternal diet was significantly higher than that of the groups supplemented with 0, 4,000, 8,000 and 16,000 IU/kg VA (p< 0.05). The IgG contents of the 8,000 IU/kg VA and 12,000 IU/kg VA groups were significantly higher than that of the group with no VA supplementation (p<0.05). The IgG content of offspring in the 9,000 IU/kg VA supplementation group was significantly higher than that in the group with no VA supplementation (p<0.05). The IgM contents of the 8,000 and 12,000 IU/kg VA supplementation groups were significantly higher than those of the 0, 4,000, and 16,000 IU/kg VA supplementation groups (p<0.05). Maternal VA supplementation level and offspring VA supplementation level had significant interaction in IgA and IgM of goslings (p<0.05).

## DISCUSSION

### Intestinal tissue morphology

The intestinal tract is the main place for digestion and the absorption of nutrients. Histomorphological observation is an important indicator of intestinal development. The length and width of the villi can reflect the area of the intestinal tract in contact with nutrients, and an increased contact area can increase nutrient absorption by the intestine [18]. The depth of the crypts can reflect the maturity of crypt cells. The maturity of crypt cells increases with decreasing crypt depth, and their secretory function is also enhanced. Muscle thickness can reflect intestinal motility. Increasing the thickness of the muscle layer will enhance intestinal motility and improve intestinal digestive function. Nzegwu et al [19] showed that the addition of VA to the diet changes the tissue morphology of the intestine. Tian et al [20] demonstrated that VA can alter the intestinal environment and immune function. Our experimental results show that the intestinal tissue morphology of goslings was the best when the maternal diet was supplemented with 12,000 IU/kg VA and the offspring diet was supplemented with 9,000 IU/kg VA. Good intestinal development can promote the growth of goslings. However, adding excessive VA to the diet can destroy the intestinal morphology of goslings.

### Immune function

Animal immune organs are the place where lymphocytes and other immune cells develop, differentiate, mature, settle, proliferate and produce an immune response. The thymus and bursa of Fabricius are important central immune organs in poultry. They develop at a young age, mature at sexual maturity, and then degenerate gradually. The spleen is the peripheral immune organ, and it survives for life. Immunoglobulin is the main effector that mediates serum immunity and plays an important role in the body’s defense system. IgG accounts for 75% to 80% of the total serum Ig content and is the main antibody produced by the immune system to fight infections. The VA acid is the intermediate product of VA metabolism. VA acid can enhance the immune function of animals by regulating T cell differentiation and cell transport [21]. Goverse et al [22] showed that VA acid is key to enhancing immunity in mice. Tan et al [23] found that VA regulates the immune system by allowing VA acid to enter the spleen quickly and be absorbed by the spleen. Mullin [24] observed that a moderate amount of VA can enhance immunity. Yuan et al [2] concluded that adding too much VA could reduce immunity in broilers. Generally, the experimental results showed that the development of immune organs in goslings was the best when 12,000 IU/kg VA was added to the maternal diet and 9,000 IU/kg VA was added to the offspring diet. An appropriate level of VA can improve the immune organ index of goslings. The serum Ig level in goslings correlated with the dietary VA level. When VA is excessive, the immune function of goslings is reduced. These results are consistent with those of previous studies and verify the above statement.

In summary, the growth and development of offspring were affected by the amount of VA added to the maternal diet. The VA can be passed on from maternal to offspring. Supplementation of 12,000 IU/kg VA in the maternal diet and 9,000 IU/kg VA in the offspring diet supports optimal gosling growth. When 16,000 IU/kg VA was supplemented in the maternal diet, the offspring did not need to be supplemented with VA. Maternal supplementation with too little VA can be corrected by adding VA to the offspring diet. Continued VA supplementation in offspring diet after excessive VA supplementation in the maternal diet is unfavorable for the growth and development of goslings.

## Figures and Tables

**Table 1:** Composition and nutrient levels of basal diets for geese and offspring (dry basis)

Item	Breeding geese	Offspring
Ingredients (%)		
Corn	63.00	63.00
Soybean meal	23.00	30.20
Rice husk	8.70	3.20
Methionine	0.26	0.10
Salt	0.30	0.30
Stone powder	2.00	1.10
Calcium hydrogen phosphate	1.50	1.10
Choline chloride	0.24	-
Premix1)	1.00	1.00
Total	100.00	100.00
Nutritional level2)		
Metabolizable energy (MJ/kg)	11.12	11.34
Crude protein (%)	15.54	18.98
Crude fiber (%)	5.86	4.07
Ca (%)	1.29	0.83
Total phosphorus (%)	0.59	0.56
Effective phosphorus (%)	0.37	0.32
Lysine (%)	0.76	0.99
Methionine (%)	0.49	0.42
Vitamin A (IU/kg)	1,210.00	1,277.00

1) Breeding geese per kilogram of premix contains: vitamin D_3_ 225,000 IU, vitamin E 3,600 IU, vitamin K 225 mg, vitamin B_1_ 220 mg, vitamin B_2_ 975 mg, vitamin B_6_ 375 mg, vitamin B_12_ 2.75 mg, nicotinic acid 3,750 mg, pantothenic acid 1,500 mg, folic acid 140 mg, biotin 9.5 mg, choline chloride 55 g, Fe 8 g, Cu 0.5 g, Mn 10 g, Zn 10 g, Se 30 mg, I 125 mg.

Offspring contain per kilogram of premix: vitamin D 400,000 IU, vitamin E 1,800 IU, vitamin K 150 mg, vitamin B_1_ 60 mg, vitamin B_2_ 600 mg, vitamin B_6_ 200 mg, vitamin B_12_ 1 mg, niacin 3,000 mg, D-pantothenic acid 900 mg, folic acid 50 mg, biotin 4 mg, choline 35 g, Fe 6 g, Cu 1 g, Mn 9.5 g, Zn 9 g, Se 30 mg, I 50 mg.

2) Vitamin A is the measured value, and the rest are calculated values.

**Table 2:** Effects of different levels of vitamin A in maternal and offspring diets on duodenal morphology in goslings (μm)

Items	Villus length	Villus width	Crypt depth	Myometrial thickness
Maternal VA level (IU/kg)				
0	579.94^b^	100.22^b^	175.6^a^	201.78^c^
4,000	585.56^b^	107.79^b^	164.49^ab^	210.73^c^
8,000	623.56^ab^	123.51^a^	157.55^ab^	243.92^ab^
12,000	682.75^a^	127.26^a^	150.31^b^	263.71^a^
16,000	649.15^ab^	100.34^b^	173.32^a^	221.66^b^
SEM	11.10	2.50	3.05	4.54
Gosling VA level (IU/kg)				
0	593.15^b^	111.30	162.32	229.95
9,000	655.24^a^	112.35	166.18	226.77
SEM	20.86	5.03	6.13	9.16
p-value				
Maternal VA level	0.012	<0.001	0.040	<0.001
Gosling VA level	0.004	0.836	0.531	0.729
Maternal VA level×gosling VA level interaction	0.002	0.794	0.522	0.640
Linear	0.002	0.184	0.363	0.001
Quadratic	0.507	<0.001	0.007	<0.001

VA, vitamin A; SEM, standard error of the mean.

The results are average values, n = 12 when the maternal VA level is the main factor, and n = 30 when the VA level of the offspring is the main factor.

a–c Means with different letters in the same colum differ at p<0.05.

**Table 3:** Effects of different vitamin A levels in maternal and offspring diets on jejunum histomorphology of goslings (μm)

Items	Villus length	Villus width	Crypt depth	Myometrial thickness
Maternal VA level (IU/kg)				
0	671.60^ab^	97.56^bc^	197.85^a^	213.58^b^
4,000	695.44^ab^	104.89^ab^	179.33^ab^	211.25^b^
8,000	722.01^a^	114.16^a^	168.66^b^	268.14^a^
12,000	720.01^a^	116.47^a^	146.84^c^	262.75^a^
16,000	650.95^b^	90.28^c^	197.55^a^	226.25^b^
SEM	9.06	2.18	3.97	5.14
Gosling VA level (IU/kg)				
0	688.87	101.99	177.67	227.04
9,000	695.03	107.35	178.42	245.75
SEM	18.26	4.34	8.00	10.07
p-value				
Maternal VA level	0.048	<0.001	<0.001	<0.001
Gosling VA level	0.737	0.221	0.926	0.068
Maternal VA level×gosling VA level interaction	0.300	0.001	0.001	0.028
Linear	0.778	0.818	0.148	0.012
Quadratic	0.004	<0.001	<0.001	<0.001

VA, vitamin A; SEM, standard error of the mean.

The results are average values, n = 12 when the maternal VA level is the main factor, and n = 30 when the VA level of the offspring is the main factor.

a–c Means with different letters in the same colum differ at p<0.05.

**Table 4:** Effects of different vitamin A levels in maternal and offspring diets on ileum histomorphology of goslings (μm)

Items	Villus length	Villus width	Crypt depth	Myometrial thickness
Maternal VA level (IU/kg)				
0	531.92^c^	79.57^c^	182.62^a^	222.16^c^
4,000	541.68^c^	84.14^bc^	155.92^b^	222.65^c^
8,000	623.64^a^	93.93^ab^	155.87^b^	284.74^a^
12,000	603.10^ab^	101.03^a^	161.61^b^	300.43^a^
16,000	562.65^bc^	87^bc^	173.20^ab^	250.96^b^
SEM	9.05	1.79	3.04	5.49
Gosling VA level (IU/kg)				
0	561.61	83.89^b^	156.61^b^	250.78
9,000	583.69	94.37^a^	175.08^a^	261.60
SEM	18.02	3.35	5.64	10.78
p-value				
Maternal VA level	0.002	<0.001	0.130	<0.001
Gosling VA level	0.225	0.003	0.002	0.328
Maternal VA level×gosling VA level interaction	0.178	<0.001	0.001	0.140
Linear	0.035	0.006	0.512	<0.001
Quadratic	0.004	0.003	0.001	<0.001

VA, vitamin A; SEM, standard error of the mean.

The results are average values, n = 12 when the maternal VA level is the main factor, and n = 30 when the VA level of the offspring is the main factor.

a–c Means with different letters in the same colum differ at p<0.05.

**Table 5:** Effects of different vitamin A levels of maternal and offspring diets on immune organ index of offspring

Items	Thymus index	Spleen index	Bursa index
Maternal VA level (IU/kg)			
0	2.66^d^	1.33^ab^	1.17^b^
4,000	3.14^cd^	1.11^c^	1.22^b^
8,000	3.39^bc^	1.11^c^	1.30^ab^
12,000	3.92^a^	1.47^a^	1.42^a^
16,000	3.80^ab^	1.28^bc^	1.45^a^
SEM	0.10	0.03	0.03
Gosling VA level (IU/kg)			
0	3.62^a^	1.30	1.29
9,000	3.15^b^	1.23	1.34
SEM	0.18	0.07	0.06
p-value			
Maternal VA level	<0.001	0.001	0.008
Gosling VA level	0.014	0.352	0.376
Maternal VA level ×gosling VA level interaction	0.002	<0.001	<0.001
Linear	<0.001	0.213	<0.001
Quadratic	0.180	0.079	0.970

VA, vitamin A; SEM, standard error of the mean.

The results are average values, n = 12 when the maternal VA level is the main factor, and n = 30 when the VA level of the offspring is the main factor.

a–d Means with different letters in the same colum differ at p<0.05.

**Table 6:** Effects of different vitamin A levels of maternal and offspring diets on immunoglobulin content of offspring in serum (ng/mL)

Items	IgA	IgG	IgM
Maternal VA level (IU/kg)			
0	0.26^c^	54.87^b^	2.67^b^
4,000	0.27^c^	59.85^ab^	2.68^b^
8,000	0.4^b^	60.61^a^	3.25^a^
12,000	0.48^a^	62.6^a^	3.43^a^
16,000	0.3^c^	58.64^ab^	2.24^c^
SEM	0.01	0.83	0.08
Gosling VA level (IU/kg)			
0	0.36	57.1^b^	2.81
9,000	0.32	61.53^a^	2.9
SEM	0.03	1.57	0.15
p-value			
Maternal VA level	<0.001	0.042	<0.001
Gosling VA level	0.14	0.007	0.522
Maternal VA level ×gosling VA level interaction	<0.001	0.954	0.007
Linear	<0.001	0.07	0.772
Quadratic	<0.001	0.014	<0.001

Ig, immunoglobulin; VA, vitamin A; SEM, standard error of the mean.

The results are average values, n = 12 when the maternal VA level is the main factor, and n = 30 when the VA level of the offspring is the main factor.

a–c Means with different letters in the same colum differ at p<0.05.
